# Dietary Fructose Activates Insulin Signaling and Inflammation in Adipose Tissue: Modulatory Role of Resveratrol

**DOI:** 10.1155/2016/8014252

**Published:** 2016-03-15

**Authors:** Mehmet Bilgehan Pektas, Halit Bugra Koca, Gokhan Sadi, Fatma Akar

**Affiliations:** ^1^Department of Medical Pharmacology, Faculty of Medicine, Afyon Kocatepe University, 03100 Afyonkarahisar, Turkey; ^2^Department of Pharmacology, Faculty of Pharmacy, Gazi University, 06330 Ankara, Turkey; ^3^Department of Medical Biochemistry, Faculty of Medicine, Afyon Kocatepe University, 03100 Afyonkarahisar, Turkey; ^4^Department of Biology, K.Ö. Science Faculty, Karamanoglu Mehmetbey University, 70100 Karaman, Turkey

## Abstract

The effects of high-fructose diet on adipose tissue insulin signaling and inflammatory process have been poorly documented. In this study, we examined the influences of long-term fructose intake and resveratrol supplementation on the expression of genes involved in insulin signaling and the levels of inflammatory cytokines and sex hormones in the white adipose tissues of male and female rats. Consumption of high-fructose diet for 24 weeks increased the expression of genes involved in insulin signaling including* IR*,* IRS-1*,* IRS-2*,* Akt*,* PI3K*,* eNOS*,* mTOR*, and* PPARγ*, despite induction of proinflammatory markers, iNOS, TNF*α*, IL-1*β*, IL-18, MDA, and ALT, as well as anti-inflammatory factors, IL-10 and Nrf2 in adipose tissues from males and females. Total and free testosterone concentrations of adipose tissues were impaired in males but increased in females, although there were no changes in their blood levels. Resveratrol supplementation markedly restored the levels of MDA, IL6, IL-10, and IL-18, as well as* iNOS*,* Nrf2*, and* PI3K* mRNA, in adipose tissues of both genders. Dietary fructose activates both insulin signaling and inflammatory pathway in the adipose tissues of male and female rats proposing no correlation between the tissue insulin signaling and inflammation. Resveratrol has partly modulatory effects on fructose-induced changes.

## 1. Introduction

The regional storage of fat tissue varies between male and female gender. Adipose tissue has a function as endocrine and metabolic organ secreting several hormones and factors that affect fat and glucose metabolism as well as insulin sensitivity [1]. Disruption of adipose tissue function and production of inflammatory cytokines may aggravate the development of metabolic disorders [2]. Moreover, the expansion of white adipose tissue was determined to be related with insulin resistance and low-grade inflammation [3]. Effect of insulin is initiated by its receptor activation through insulin receptor substrates (IRS-1 and IRS-2), which triggered downstream signaling pathways in adipocytes. In obesity, macrophages infiltrate adipose tissue and begin to induce proinflammatory cytokines, such as interleukin-1 beta (IL-1*β*), tumor necrosis factor alpha (TNF*α*), and interleukin-6 (IL-6), which can interfere with insulin signaling in adipocytes [4]. However, little is known about the influence of dietary components, such as fructose, on visceral fat accumulation and its function. High-fat diet feeding was found to cause early onset of insulin resistance and activation of inflammatory process particularly in vasculature and then in skeletal muscle and liver but lately in adipose tissue indicating a less sensitivity of fat tissue to detrimental effects of nutritional factors [5]. Moreover, high-fructose diet in rats has been reported to activate the inflammatory factors such nuclear factor kappa B (NF*κ*B) and TNF*α* in the liver, but not in the adipose tissue, showing its tissue-specific effects [6]. In this sense, we have shown that dietary high-fructose corn syrup (HFCS) for 12 weeks caused early vascular injury and insulin resistance, but there was a subinflammatory state in the liver, despite increased hepatic lipogenesis [7, 8]. The mechanism of insulin resistance and its relation to inflammatory process in the different tissues, especially in adipose tissue, are poorly understood [9].

The growing global epidemic of metabolic syndrome may be related with an excessive consumption of fructose in current human diet, particularly in the form of sweetened beverages. Some evidence supports that estrogen protects females from the signs of diet-induced metabolic disturbances [10–12]. However, the differential disease susceptibility depending on diet composition between males and females is not well investigated and experimental metabolic syndrome studies are generally performed on male animals. High-fructose diet is well documented to provoke metabolic disturbances in male rats; however, it remains to be established if there are differences in adipose tissue reactivity to dietary fructose between males and females. Recently, we suggested that fructose-induced metabolic dysfunction could be related with abdominal fat accumulation, but independent of the general obesity, in the females, differently from the males [13]. Furthermore, in the above study, we also showed that resveratrol, a multifunctional compound, which is found in grape and wine, leads to a significant decrease in omental weight in association with the improvement of hyperinsulinemia and hypertriglyceridemia in male and female rats upon fructose feeding. The investigation of effects of high-fructose diet on adipose tissue insulin signaling and inflammatory process and their modification by resveratrol will provide new insights to understand the mechanisms. Therefore, herein, we investigated the effects of dietary fructose and resveratrol supplementation on gene expressions of insulin signaling elements and inflammatory cytokines in adipose tissue of male and female rats. Thus, in this study with long-term high-fructose diet (for 24 weeks, 10% beverage), we aimed to make a simulation for the consumption of current high-carbohydrate diet in human subjects.

## 2. Materials and Methods

### 2.1. Chemicals

Chemicals were purchased from Sigma Chemical Co. (St. Louis, MO) unless otherwise stated. Fructose was obtained from Danisco Sweeteners OY (Finland) and* trans*-resveratrol was from Herb-Tech (ROC). The purity of resveratrol was tested by HPLC followed with LC-MS and 98% of the constituent was determined as* trans*-resveratrol.

### 2.2. Animals and Diets

The animal protocols were approved by the Ethical Animal Research Committee of Gazi University (GU ET-10.045). Four-week-old male and female Wistar rats were housed under temperature- and humidity-controlled rooms (20–22°C) with a 12 h light-dark cycle. The animals were fed with a standard rodent chow diet that was composed of 62% starch, 23% protein, 4% fat, 7% cellulose, standard vitamins, and salt mixture. After acclimation for 1 week, male and female rats were randomly divided into four groups as control, resveratrol, fructose, and resveratrol plus fructose (resveratrol + fructose). Fructose was given to the rats as 10% solution in drinking water* ad libitum* for 24 weeks. Resveratrol was added to chow at a dose of 500 mg/kg, which was kept under protection from light. All rats were fed with the standard diet with or without resveratrol* ad libitum* for 24 weeks. Body weights and food and liquid intakes were recorded weekly during the follow-up period. The daily resveratrol ingestion was calculated from the amount of chow intake. The daily fructose consumption was determined by measuring the liquid intake. The female rats were randomly chosen on different days of the estrous cycle on the time of sacrifice. At the end of the follow-up period, the rats were anesthetized with a mixture of ketamine-xylazine (100 and 10 mg/kg, resp., i.p.) and thereafter, blood samples were rapidly collected via cardiac puncture. The omental adipose tissues were dissected, blotted dry, weighed, and frozen in liquid nitrogen and stored at −85°C.

### 2.3. Measurement of Metabolic Parameters in the Plasma and Adipose Tissue

Cardiac blood samples of nonfasted male and female rats were immediately centrifuged at 4°C and 10,000 g for 30 min. Adipose tissue samples were homogenized with 0.1 M phosphate buffer 1 : 10 (w/v), pH 7.4, and 24,000 cycles/min (Ultra Turrax, USA) and then ultrasonicated at 20,000 cycles/sec for 1 min (Dr. Hielscher, Germany). Homogenates were centrifuged at 4°C at 10,000 g for 15 min and the supernatants were collected. All samples were stored at −85°C until analysis.Plasma triglyceride levels and alanine aminotransferase (ALT) and aspartate transaminase (AST) activities were determined by using standard enzymatic techniques (Biolabo, France). Insulin (Mercodia, Sweden), estradiol, free and total testosterone, TNF*α*, IL-1*β*, IL-6 and interleukin-10 (IL-10) (eBioscience, USA), interleukin-18 (IL-18) levels (Cusabio, China) were measured by using commercial ELISA kits according to the manufacturer's instructions. Malondialdehyde (MDA) levels were measured with thiobarbituric acid reactive substances (TBARS) assay kit (Cayman Chemical, USA).

### 2.4. Determination of the Gene Expressions with Real Time Polymerase Chain Reaction

Total RNAs were isolated from the abdominal tissues using RNeasy total RNA isolation kit (Qiagen, Venlo, Netherlands) as described according to the manufacturer protocol. After isolation, the amount and the quality of the total RNAs were determined by spectrophotometry and agarose gel electrophoresis. Then, 1 *µ*g of total RNA was reverse-transcribed to cDNA using commercial first-strand cDNA synthesis kit (Thermo Scientific, USA). Expression levels of insulin receptor beta (*IRβ*),* IRS-1*,* IRS-2*, protein kinase B (*Akt*), phosphoinositide 3-kinase (*PI3K*), endothelial nitric oxide synthase (*eNOS*), sirtuin 1 (*SIRT1*), mammalian target of rapamycin (*mTOR*), peroxisome proliferator-activated receptor gamma (*PPARγ*), nuclear factor erythroid 2-related factor 2 (*Nrf2*), nuclear factor kappa B (NF*κ*B), and inducible nitric oxide synthase (*iNOS*) genes were determined with real-time polymerase chain reaction (qRT-PCR, LightCycler480 II, Roche, Basel, Switzerland). To do this, 1 *μ*L cDNA, 5 *μ*L 2X SYBR Green Master Mix (Roche FastStart Universal SYBR Green Master Mix), and primer pairs (Table 1) at 0.5 *µ*M concentrations in a final volume of 10 *µ*L were mixed and qRT-PCR was performed as follows: initial denaturation at 95°C for 10 minutes, denaturation at 95°C for 10 seconds, annealing at 58°C for 15 seconds, and extension at 72°C for 15 seconds with 45 repeated thermal cycles measuring the green fluorescence at the end of each extension step. All reactions were performed in triplicate and the specificity of PCR products was confirmed using melt analysis. The relative expression of genes with respect to internal control glyceraldehyde 3-phosphate dehydrogenase (*GAPDH*) was calculated with the efficiency corrected advance relative quantification tool provided by the LightCycler® 480 SW 1.5.1 software.

### 2.5. Statistical Analysis

All data are given as mean ± standard error of the mean; *n* is the number of rats. Statistical comparisons were performed by using unpaired Student's *t*-test or one-way ANOVA followed by the Bonferroni* post hoc* test. *P* values smaller than 0.05 were considered as statistically significant.

## 3. Results

### 3.1. The Effects of Dietary Fructose and Resveratrol on Metabolic and Endocrine Parameters in the Plasma and the Adipose Tissues

The data representing body weight, omental fat mass, daily food, and liquid and caloric intakes, as well as resveratrol and fructose ingestions of rats, have been published in our recent study [13]; however, they were again included in Table 2 to facilitate understanding of the current study. Dietary fructose caused a significant body weight gain in male rats (*P* < 0.05 versus corresponding control), which is reduced with resveratrol supplementation (*P* < 0.05 versus corresponding fructose), but not in female rats (Table 2). Importantly, fructose treatment augmented omental fat mass in both male and female rats (*P* < 0.05 versus their corresponding control groups), which responded to resveratrol supplementation with a significant reduction (*P* < 0.05 versus their corresponding fructose groups, Table 2). High-fructose diet increased plasma triglyceride and insulin levels (*P* < 0.05 versus their corresponding control groups), which are significantly reduced by resveratrol (*P* < 0.05 versus their corresponding fructose groups), in both genders (Table 2) as demonstrated in our recent study [13]. Resveratrol decreased insulin level in adipose tissue of healthy female rats (*P* < 0.05 versus corresponding control). Dietary fructose increased insulin level in adipose tissue of both male and female rats (*P* < 0.05 versus their corresponding control groups), which responded to resveratrol supplementation with a significant reduction (*P* < 0.05 versus their corresponding fructose groups, Table 3).

Resveratrol decreased free testosterone level in adipose tissue but increased in plasma of healthy female rats (*P* < 0.05 versus corresponding control). Dietary fructose did not affect the plasma level of testosterone in both genders; however, this dietary intervention impaired the total and free testosterone concentrations of adipose tissue in males (*P* < 0.05 versus corresponding control) and contrarily increased those of females (*P* < 0.05 versus corresponding control). Moreover, fructose feeding decreased estrogen level in adipose tissue of females (*P* < 0.05 versus corresponding control), without changing in that of males. Resveratrol supplementation diminished free and total testosterone levels in adipose tissue of females upon fructose feeding (*P* < 0.05 versus corresponding fructose, Tables 2 and 3).

### 3.2. The Effects of Dietary Fructose and Resveratrol on Cytokines and Oxidative Stress Markers in the Adipose Tissues

Results show that female control rats have significantly lower IL-1*β*, IL-6, and TNF*α* levels as compared to the males, but there was an opposite condition for AST activity (*P* < 0.05). Resveratrol decreased MDA, ALT, and AST levels in adipose tissue of healthy female rats, but only IL-1*β* in those of males (*P* < 0.05 versus their corresponding controls, Table 3). High-fructose diet caused marked elevation in MDA, ALT, and AST levels in adipose tissues of male and female rats (*P* < 0.05 versus their corresponding controls). Inflammatory cytokines, TNF*α*, IL-1*β*, IL-6, IL-10, and IL-18 levels were also elevated in adipose tissues of male and female rats by dietary fructose intervention (*P* < 0.05 versus their corresponding controls). Resveratrol supplementation significantly reduced MDA, IL-6, IL-10, and IL-18 levels in adipose tissues from male and female rats upon fructose feeding (*P* < 0.05 versus their corresponding fructose groups). Moreover, this supplementation also decreased ALT, AST, and TNF*α* levels in the females (*P* < 0.05 versus corresponding fructose group, Table 3).

### 3.3. The Effects of Dietary Fructose and Resveratrol on IR*β*, IRS-1, IRS-2, Akt, PI3K, eNOS, SIRT1, mTOR, PPAR*γ*, Nrf2, *NFκB*, and iNOS Gene Expressions in the Adipose Tissues

**Figure 1:**
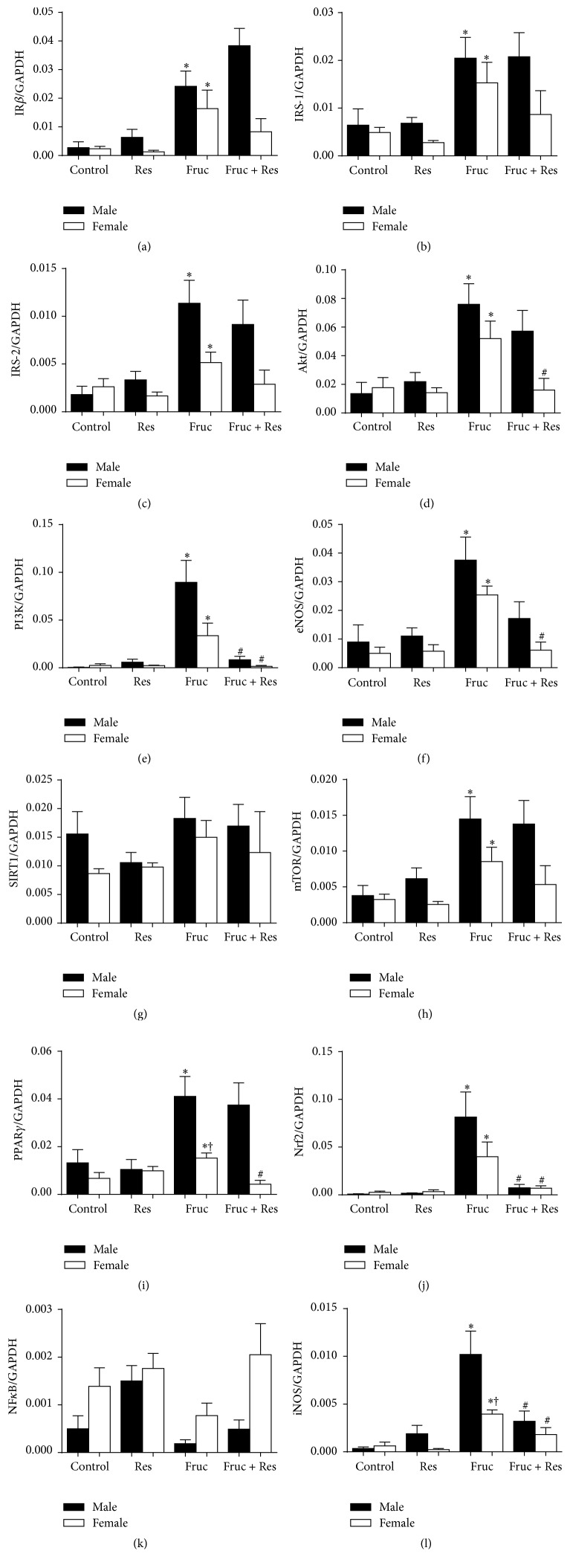
The mRNA expression levels of* IRβ* (a),* IRS-1* (b),* IRS-2* (c),* Akt* (d),* PI3K* (e),* eNOS* (f),* SIRT1* (g),* mTOR* (h),* PPARγ* (i),* Nrf2* (j), *NFκB* (k), and* iNOS* (l) in the adipose tissues of male and female rats from the control, resveratrol (Res), fructose (Fruc), and Res + Fruc groups. Data was normalized by* GAPDH*. Each bar represents the means of at least six rats. Values are expressed as mean ± SEM, *n* = 6–12. ^*∗*^Significantly different (*P* < 0.05) compared to control group; ^#^significantly different (*P* < 0.05) compared to fructose group; ^†^significantly different (*P* < 0.05) compared to male group.

The gene expression levels of* IRβ*,* IRS-1*,* IRS-2*,* Akt*,* PI3K*,* eNOS*,* SIRT1*,* mTOR*,* PPARγ*,* Nrf2*, *NFκB*, and* iNOS* in the adipose tissue samples from male and female rats were established by real-time PCR analysis. Dietary fructose increased* IRβ*,* IRS-1*,* IRS-2*,* Akt*,* PI3K*,* eNOS*,* mTOR*,* PPARγ*,* Nrf2*, and* iNOS* mRNA expressions in the adipose tissue samples from male and female rats (*P* < 0.05 versus their corresponding control groups), whereas no changes were observed in *NFκB* and* SIRT1* mRNA expressions of both genders (Figures 1(a)–1(l)). There was a blunted increase in* iNOS* and* PPARγ* mRNA levels in the adipose tissue of female rats in response to dietary fructose (*P* < 0.05 versus their corresponding males). However, taken all together, being female does not provide any protection against harmful effects of fructose.

Resveratrol supplementation significantly reduced* PI3K* and* iNOS* mRNA expressionsin male and female rats upon fructose feeding. However, this supplementation decreased* Akt*,* PPARγ*, and* eNOS* mRNAs only in the females, and* Nrf2* mRNAin the males (*P* < 0.05 versus their corresponding fructose groups, Figures 1(a)–1(l)).

## 4. Discussion

A characteristic feature of metabolic syndrome is enlargement of visceral adipose tissue. In a very recent study, we have shown that dietary fructose causes an increase in the plasma level of insulin and triglyceride, in association with expansion of omental mass, pointing metabolic syndrome and increased visceral adiposity in male and female rats [13]. The effect of high-fructose diet on adipose tissue insulin signaling and its relation to inflammatory process are not well characterized. Extending our study, herein, we focused on the influence of long-term fructose intake in the expression of genes involved in insulin signaling and inflammatory cytokines in the adipose tissue of rats of both genders. Our findings showed that dietary fructose-induced visceral fat accumulation led to increased expression of genes functioning in insulin signaling, changed endocrine function, and activated proinflammatory and anti-inflammatory markers, in adipose tissue from male and female rats. Gender-dependent differences to fructose feeding were not prominent suggesting that being female does not provide any advantage in protection from harmful effects of fructose. Resveratrol supplementation restored the increased levels of MDA, IL-10, and IL-18 as well as expression of* iNOS* and* PI3K* mRNAs in adipose tissue from both genders; however, its effectiveness on the insulin signaling pathway and other parameters measured was limited or rather gender-dependent.

Our recent findings revealed that dietary fructose-induced metabolic disorder is more likely linked to abdominal fat accumulation, but independent of the general obesity. Long-term dietary fructose increased body weight of males, whereas it did not change that of females [13]. Visceral adipose tissue mass was increased in subjects consuming fructose-sweetened beverages, not those consuming glucose-sweetened beverages, suggesting that dietary fructose is more closely associated with metabolic disease and adiposity [14]. Insulin signaling in adipocytes is initiated by its receptor activation through IRS-1 and IRS-2 transmitting signal to the intracellular effectors. Insulin signaling also elongates various links to eNOS, SIRT1, PPAR*γ*, and mTOR pathways [9, 15–17]. Insulin may activate lipogenic genes and stimulate lipogenesis in adipocytes, recognized as* de novo* fatty acid synthesis, but the mechanisms are less known than those of liver [9]. In the liver, high-fructose diet causes hyperinsulinemia and increased lipogenesis which in turn raises ectopic lipid deposition, despite hepatic insulin resistance and inadequate control of hyperglycemia [8, 18, 19]. High-fat and fructose diets were shown to stimulate lipogenesis by increasing FAS gene expression in adipose tissue of mice [20–22]. Differently, it has been reported that the expression of lipogenic transcription factor SREBP1 and lipogenesis are decreased in adipose tissue of obese mice, although expression of inflammatory genes in adipocytes and hepatic lipogenic capacity are increased [23, 24]. These results revealed that lipogenic response in adipose tissue in the metabolic syndrome and obesity has not yet been entirely understood.

Previously, it has been shown that disruption of IRS-2 in mice caused a marked insulin resistance in adipose tissue [25]. Phosphorylation of IRS-1 and IRS-2 was detected to be increased in adipose tissue lysates of hyperinsulinemic mice [20]. Herein, we detected an elevation in adipose tissue level of insulin and upregulation of* IRβ*,* IRS-1*, and* IRS-2* mRNAs, as well as insulin downstream effectors* Akt*,* PI3K*,* eNOS*,* mTOR*, and* PPARγ* mRNAs, but no change in* SIRT1* mRNA expression, in association with expansion of omental mass of male and female rats. This could be a differing situation to those seen in other insulin sensitive organs such as liver, muscle, and vascular system, where downregulation of insulin signaling had been determined as a consequence of fructose consumption in rodents [7, 8, 13, 26, 27]. It is therefore tempting to propose that the upregulation of insulin signaling pathway in adipose tissue leads to the increased visceral adiposity. In studies investigating dietary fructose on fat tissue, short-term fructose solution drinking (10% fructose for 3 weeks) was found to decrease the expression of IRS-1 and IRS-2 genes in enlarged abdominal adipose tissue of rats [28, 29]. Consumption of high-fructose corn syrup (for 10 months) caused downregulation of IRS-1 gene in association with increased expression of TNF*α*, IL-1*β*, and IL-6 in intra-abdominal adipose tissue, but not in those of liver and skeletal muscle, in mice [30]. These tissue-specific and contradictory results warrant additional studies to understand the accurate influence of fructose given in diet on the insulin effectors in adipose and other sensitive organs.

In obesity, macrophages infiltrate adipose tissue and begin to induce proinflammatory cytokines, such as IL-1*β*, TNF*α*, and IL-6, which contribute to insulin resistance [4]. Long-term treatment of adipocytes with IL-6, IL-1*β*, or TNF*α* was shown to inhibit insulin signaling and cause insulin resistance [31, 32]. High-fructose diet in rats has been reported to activate the inflammatory factors including NF-*κ*B and TNF*α* in the liver, differently from the adipose tissue, signifying tissue-specific effects of dietary fructose [6]. Herein, dietary fructose increased the levels of proinflammatory markers, iNOS, TNF*α*, IL-1*β*, IL-18, MDA, and ALT, as well as anti-inflammatory factors, IL-10, Nrf2, and mTOR, in adipose tissues from males and females. These latter factors could be activated as a compensatory mechanism to counteract inflammation as reported previously in adipose tissue [16, 33, 34]. In this sound, mRNA level of PPAR*β*/*δ*, which is an insulin sensitizing molecule, was found to be increased in visceral adipose tissue from insulin-resistant patients, in spite of the presence of inflammatory condition, proposing a compensatory mechanism [35], in accordance with our results with increased expression of* PPARγ* mRNA due to fructose diet. A recent study showed that fructose-enriched diet (for 8 weeks) activates proinflammatory cytokine IL-6, accompanied by a reduction of PPAR*γ*, without causing hyperinsulinemia, in adipose tissue of rats [36]. In a related study, it has been shown that high-fructose diet lowered IL-10, but did not change TNF*α* levels; however, PPAR*β*/*δ* deficiency made the insulin resistance apparent, in association with increased inflammatory markers, in adipose tissue of mice [37]. The discrepancy between the aforementioned and the present studies can be ascribed to the differences in the method and feeding duration to induce metabolic disorder. Regarding gender differences to fructose diet, all our above results indicated that being female does not provide any advantage in protection from harmful effects of this nutritional intervention.

Our data with sex hormones demonstrated that dietary fructose changes endocrine function of adipose tissues of rats, in which total and free testosterone levels were impaired in males, but increased in females. Differently, estrogen level was decreased in the females without changing in the males. There was no change in the blood levels of testosterone and estrogen, which is partly consistent with previous results in high-fructose-fed rats [38, 39]. The significance of reciprocal adverse effect of dietary fructose on testosterone levels of males and females, as well its diminishing effect on estrogen of females, in the adipose tissues remains to be understood, which could be an imperative issue. On the other hand, restoring effect of resveratrol on testosterone level in adipose tissue of females may have a potential interest.

Resveratrol supplementation did not change the increased* IRβ*,* IRS-1*, and* IRS-2* mRNA expressions in adipose tissue of high-fructose-fed male and female rats. However, we found a decrease in* PI3K* mRNA in both genders, as well as in* Akt*,* eNOS*, and* PPARγ* mRNA expressions in females after resveratrol supplementation. It was previously reported that resveratrol treatment restored the insulin-stimulated Akt (Ser^473^) phosphorylation in liver and adipose tissue, but not in the skeletal muscle, of high-fat-fed mice [40]. In high-fat, high-sugar diet-fed rhesus monkeys, resveratrol supplementation was shown to increase IRS-1 protein levels but decrease Akt (Ser^473^) phosphorylation in visceral adipose tissue [41]. In that study, resveratrol was also demonstrated to increase SIRT1 expression in visceral, but not subcutaneous, adipose tissue. Herein,* SIRT1* mRNA level was not changed with resveratrol treatment in both genders. In our recent studies, resveratrol supplementation increased* IRS-1* mRNA and protein as well as* IRS-2* mRNA levels in liver, but not in vascular tissue, of rats fed with HFCS [7, 8]. However, in a comparison study between genders, we determined some differences on vascular upregulation of the IRS-1 of males and IRS-2 of females upon fructose feeding by resveratrol treatment [13]. Thus, it can be assumed that diversity in the effects of resveratrol on insulin signaling pathway may depend on the types of tissues, animals, and diets as well as gender.

A decrease in MDA, IL-6, IL-10, and IL-18 levels as well as in* Nrf2* and* iNOS* mRNAs was observed after resveratrol supplementation to fructose feeding male and female rats. Additionally, TNF*α*, AST, and ALT levels were reduced in females which may have a gender-dependent response to resveratrol. These findings are consistent with earlier observations showing that resveratrol suppressed inflammatory cytokine expression and oxidative stress markers in adipose tissue of rats [42] and epididymal fat tissues of mice [5] and adipose tissue of rhesus monkeys [41]. Current findings suggested that resveratrol could be more effective on inflammatory parameters in adipose tissue from females than those of males upon fructose feeding. Regarding gender differences, resveratrol decreased insulin, free testosterone, MDA, and ALT and AST levels in adipose tissue of healthy female rats, but only IL-1*β* in those of males. These findings also showed that resveratrol has gender-dependent potential.

In conclusion, dietary fructose-induced gene expression in insulin signaling pathway, in association with the activation of inflammatory markers, led us to propose that there could be no correlation between insulin signaling and inflammation in adipose tissue of male and female rats. Resveratrol has limited modulatory effects on these unexpected changes. Further studies are necessary to clarify the relationship between insulin signaling pathway and inflammation in adipose tissue due to fructose diet.

## Figures and Tables

**Table 1:** Primer sequences of *IRβ*, *IRS-1*, *IRS-2*, *Akt*, *PI3K*, *eNOS*, *SIRT1*, *mTOR*, *PPARγ*, *Nrf2*, *NFκB*, *iNOS*, and internal standard *GAPDH* used for the mRNA expression determination of qRT-PCR.

Gene	Forward primer sequence (5′ → 3′)	Reverse primer sequence (5′ → 3′)	Product length (bp)	Gene bank accession number
*IRβ*	GTGCTGCTCATGTCCTTAGA	AATGGTCTGTGCTCTTCGTG	234	XM_006248753.2
*IRS-1*	GCCAATCTTCATCCAGTTGC	CATCGTGAAGAAGGCATAGG	337	NM_012969.1
*IRS-2*	CTACCCACTGAGCCCAAGAG	CCAGGGATGAAGCAGGACTA	151	NM_001168633.1
*Akt*	GAAGAAGAGCTCGCCTCCAT	GAAGGAGAAGGCCACAGGTC	211	NM_033230.2
*PI3K*	ATGCAACTGCCTTGCACATT	CGCCTGAAGCTGAGCAACAT	320	NM_053481.2
*eNOS*	TGCACCCTTCCGGGGATTCT	GGATCCCTGGAAAAGGCGGT	189	XM_006235872.1
*SIRT1*	CGGTCTGTCAGCATCATCTTCC	CGCCTTATCCTCTAGTTCCTGTG	136	XM_008772947.1
*mTOR*	GCAATGGGCACGAGTTTGTT	AGTGTGTTCACCAGGCCAAA	94	NM_019906.1
*PPARγ*	CTCAGGTCAGAGTCGCCCC	GAGAGAGACCTCGTCAGGCT	205	NM_001145367.1
*Nrf2*	GATTCGTGCACAGCAGCA	GCCAGCTGAACTCCTTAGAC	466	XM_006234397.2
*NFκB*	GGGTCAGAGGCCAATAGAGA	CCTAGCTTTCTCTGAACTGCAAA	71	AF_079314.1
*iNOS*	CTTCAGGTATGCGGTATTGG	CATGGTGAACACGTTCTTGG	352	XM_006246949.2
*GAPDH*	TGATGACATCAAGAAGGTGGTGAAG	TCCTTGGAGGCCATGTGGGCCAT	240	NM_017008.4

**Table 2:** Effects of long-term dietary fructose (Fruc), resveratrol (Res), and their combinations (Res + Fruc) on some metabolic parameters and plasma insulin, triglyceride, estradiol, and free testosterone and total testosterone of male and female rats.

Groups		Control	Res	Fruc	Res + Fruc
Terminal body weight (g)	Male	370 ± 10.7	335 ± 4.1^*∗*^	415 ± 6.5^*∗*^	371 ± 5.1^#^
	Female	249 ± 6.7^†^	227 ± 7.5^†^	260 ± 1.8^†^	235 ± 1.5^#^

Food intake (g/day)	Male	20.74 ± 0.83	24.6 ± 1.1	20.5 ± 1.1	21.9 ± 1.1
	Female	14.4 ± 0.8^†^	18.3 ± 0.6^*∗*†^	12.7 ± 0.5^†^	12.8 ± 0.4^†^

Liquid intake (mL/day)	Male	46.5 ± 2.1	61.2 ± 2.4^*∗*^	56.8 ± 1.6^*∗*^	55.9 ± 1.6
	Female	32.9 ± 0.9^†^	38.9 ± 1.3^*∗*†^	38.1 ± 1.3^†^	37.8 ± 1.3^†^

Total caloric intake (kcal)	Male	72.6 ± 2.9	86.1 ± 3.8^*∗*^	94.9 ± 4.2^*∗*^	99.5 ± 4.5
	Female	50.5 ± 2.9^†^	64.1 ± 2.2^*∗*†^	60.1 ± 2.2^*∗*†^	60.3 ± 2.1^†^

Resveratrol intake (mg/kg bw)	Male	—	36 ± 1.2	—	29 ± 0.8
	Female	—	40 ± 2.1	—	27 ± 0.5

Fructose intake (g/day)	Male	—	—	5.7 ± 0.2	5.6 ± 0.2
	Female	—	—	3.8 ± 0.1	3.8 ± 0.1

Omentum weight/body weight (%)	Male	0.53 ± 0.02	0.57 ± 0.03	1.39 ± 0.32^*∗*^	0.95 ± 0.10^#^
	Female	0.88 ± 0.04^†^	0.59 ± 0.03^*∗*^	1.64 ± 0.23^*∗*^	1.20 ± 0.09^#^

Glucose (mg/dL)	Male	129.5 ± 10.9	95.3 ± 6.1^*∗*^	129.4 ± 19.1	113.8 ± 8.6
	Female	103.7 ± 3.7	87.6 ± 4.2	113.2 ± 7.8	100.9 ± 2.3

Insulin (ng/mL)	Male	0.48 ± 0.13	0.40 ± 0.09	4.38 ± 0.52^*∗*^	1.28 ± 0.27^#^
	Female	0.33 ± 0.08	0.29 ± 0.05	4.97 ± 0.34^*∗*^	2.08 ± 0.66^#^

Triglyceride (mg/dL)	Male	88.9 ± 9.4	49.2 ± 4.6^*∗*^	132.8 ± 8.5^*∗*^	57.4 ± 6.9^#^
	Female	81.6 ± 8.4	64 ± 8.3^*∗*^	205.1 ± 27.2^*∗*†^	102.9 ± 14.1^#†^

Estradiol (pg/mL)	Male	11.7 ± 1.3	9.3 ± 1.1	13 ± 2.3	9.9 ± 1.2
	Female	41.6 ± 13^†^	49.2 ± 10.6^†^	33.8 ± 8.7^†^	43.1 ± 6.9^†^

Free testosterone (pg/mL)	Male	25.7 ± 0.6	23.1 ± 0.9	23.1 ± 1.9	29.3 ± 1^#^
	Female	2.5 ± 0.17^†^	3.1 ± 0.09^*∗*†^	2.9 ± 0.09^†^	2.7 ± 0.02^†^

Total testosterone (ng/mL)	Male	3 ± 0.01	3.1 ± 0.12	2.7 ± 0.12	3 ± 0.1^#^
	Female	0.29 ± 0.01^†^	0.29 ± 0.01^†^	0.3 ± 0.01^†^	0.27 ± 0.02^†^

Values are expressed as mean ± SEM, *n* = 6–12.

^*∗*^Significantly different (*P* < 0.05) compared to control group.

^#^Significantly different (*P* < 0.05) compared to fructose group.

^†^Significantly different (*P* < 0.05) compared to male group.

**Table 3:** Effects of long-term dietary fructose (Fruc), resveratrol (Res), and their combinations (Res + Fruc) on some endocrine parameters and cytokines in the adipose tissues of male and female rats.

Groups		Control	Res	Fruc	Res + Fruc
Insulin (mU/g protein)	Male	1,54 ± 0.16	1.19 ± 0.17	2.75 ± 0.42^*∗*^	1.32 ± 0.27^#^
	Female	1.45 ± 0.16	0.79 ± 0.1^*∗*^	2.93 ± 0.26^*∗*^	1.63 ± 0.26^#^

Estradiol (pg/g protein)	Male	9.1 ± 0.6	8.6 ± 1.1	13 ± 1.5	9 ± 1.1
	Female	27.5 ± 8^†^	28 ± 5.1^†^	14 ± 1.8^*∗*^	22 ± 4.6^†^

Free testosterone (pg/g protein)	Male	45 ± 4.9	48 ± 7.8	21 ± 2.8^*∗*^	26 ± 4
	Female	2.8 ± 0.8^†^	1.5 ± 0.1^*∗*†^	5.6 ± 0.1^*∗*†^	2 ± 0.4^#†^

Total testosterone (pg/g protein)	Male	655 ± 51	581 ± 78	285 ± 33^*∗*^	332 ± 43
	Female	32 ± 6.1^†^	16 ± 3^†^	68 ± 9.5^*∗*†^	25.7 ± 4.9^#†^

MDA (*µ*mol/g protein)	Male	16 ± 1.9	18 ± 4.9	26 ± 3.6^*∗*^	14 ± 1.9^#^
	Female	14 ± 2.1	5.3 ± 0.8^*∗*†^	48 ± 13^*∗*†^	12 ± 2.1^#^

ALT (U/g protein)	Male	169 ± 14	192 ± 22	335 ± 73^*∗*^	448 ± 135
	Female	272 ± 47	113 ± 13^*∗*†^	736 ± 145^*∗*†^	315 ± 39^#^

AST (U/g protein)	Male	85 ± 5	119 ± 27	123 ± 28	271 ± 55^#^
	Female	164 ± 18^†^	51 ± 11^*∗*†^	346 ± 56^*∗*†^	142.1 ± 5^#†^

TNF-*α* (ng/g protein)	Male	153 ± 10	163 ± 7	261 ± 31^*∗*^	218 ± 33
	Female	111 ± 9^†^	99 ± 6^†^	319 ± 41^*∗*^	184 ± 38^#^

IL-1*β* (ng/g protein)	Male	382 ± 30	250 ± 17^*∗*^	496 ± 38^*∗*^	548 ± 44
	Female	241 ± 26^†^	270 ± 17	703 ± 108^*∗*^	445 ± 68

IL-6 (ng/g protein)	Male	61 ± 4.6	46 ± 2.5	67 ± 6.4	36 ± 5.3^#^
	Female	13 ± 1.3^†^	11 ± 1.9^†^	51 ± 7.4^*∗*^	20 ± 3.1^#†^

IL-10 (ng/g protein)	Male	50 ± 7.6	52 ± 4.7	83 ± 13.3^*∗*^	40 ± 5.8^#^
	Female	52 ± 6.2	33 ± 4.1^†^	93 ± 7.6^*∗*^	55 ± 6.1^#^

IL-18 (MBL) (*µ*g/g protein)	Male	9.4 ± 1.6	7.6 ± 0.7	39 ± 7.7^*∗*^	19 ± 3.8^#^
	Female	7.5 ± 1.4	8.3 ± 0.6	37 ± 6.2^*∗*^	14 ± 2.5^#^

Values are expressed as mean ± SEM, *n* = 6–12.

^*∗*^Significantly different (*P* < 0.05) compared to control group.

^#^Significantly different (*P* < 0.05) compared to fructose group.

^†^Significantly different (*P* < 0.05) compared to male group.

## References

[1] Scherer P. E. (2006). Adipose tissue: from lipid storage compartment to endocrine organ. *Diabetes*.

[2] Torres-Leal F. L., Fonseca-Alaniz M. H., Rogero M. M., Tirapegui J. (2010). The role of inflamed adipose tissue in the insulin resistance. *Cell Biochemistry and Function*.

[3] Bastard J.-P., Maachi M., Lagathu C. (2006). Recent advances in the relationship between obesity, inflammation, and insulin resistance. *European Cytokine Network*.

[4] Ballak D. B., Stienstra R., Tack C. J., Dinarello C. A., van Diepen J. A. (2015). IL-1 family members in the pathogenesis and treatment of metabolic disease: focus on adipose tissue inflammation and insulin resistance. *Cytokine*.

[5] Kim S., Jin Y., Choi Y., Park T. (2011). Resveratrol exerts anti-obesity effects via mechanisms involving down-regulation of adipogenic and inflammatory processes in mice. *Biochemical Pharmacology*.

[6] Veličković N., Djordjevic A., Vasiljević A., Bursać B., Milutinović D. V., Matić G. (2013). Tissue-specific regulation of inflammation by macrophage migration inhibitory factor and glucocorticoids in fructose-fed Wistar rats. *British Journal of Nutrition*.

[7] Babacanoglu C., Yildirim N., Sadi G., Pektas M. B., Akar F. (2013). Resveratrol prevents high-fructose corn syrup-induced vascular insulin resistance and dysfunction in rats. *Food and Chemical Toxicology*.

[8] Sadi G., Ergin V., Yilmaz G. (2015). High-fructose corn syrup-induced hepatic dysfunction in rats: improving effect of resveratrol. *European Journal of Nutrition*.

[9] Czech M. P., Tencerova M., Pedersen D. J., Aouadi M. (2013). Insulin signalling mechanisms for triacylglycerol storage. *Diabetologia*.

[10] Horton T. J., Gayles E. C., Prach P. A., Koppenhafer T. A., Pagliassotti M. J. (1997). Female rats do not develop sucrose-induced insulin resistance. *American Journal of Physiology—Regulatory Integrative and Comparative Physiology*.

[11] Gómez-Pérez Y., Amengual-Cladera E., Català-Niell A. (2008). Gender dimorphism in high-fat-diet-induced insulin resistance in skeletal muscle of aged rats. *Cellular Physiology and Biochemistry*.

[12] Stubbins R. E., Holcomb V. B., Hong J., Núñez N. P. (2012). Estrogen modulates abdominal adiposity and protects female mice from obesity and impaired glucose tolerance. *European Journal of Nutrition*.

[13] Pektas M. B., Sadi G., Akar F. (2015). Long-Term Dietary Fructose Causes Gender-Different Metabolic and Vascular Dysfunction in Rats: Modulatory Effects of Resveratrol. *Cellular Physiology and Biochemistry*.

[14] Stanhope K. L., Schwarz J. M., Keim N. L. (2009). Consuming fructose-sweetened, not glucose-sweetened, beverages increases visceral adiposity and lipids and decreases insulin sensitivity in overweight/obese humans. *The Journal of Clinical Investigation*.

[15] Yoshizaki T., Milne J. C., Imamura T. (2009). SIRT1 exerts anti-inflammatory effects and improves insulin sensitivity in adipocytes. *Molecular and Cellular Biology*.

[16] Zoncu R., Efeyan A., Sabatini D. M. (2011). MTOR: from growth signal integration to cancer, diabetes and ageing. *Nature Reviews Molecular Cell Biology*.

[17] Muniyappa R., Sowers J. R. (2013). Role of insulin resistance in endothelial dysfunction. *Reviews in Endocrine and Metabolic Disorders*.

[18] Brown M. S., Goldstein J. L. (2008). Selective versus total insulin resistance: a pathogenic paradox. *Cell Metabolism*.

[19] Dekker M. J., Su Q., Baker C., Rutledge A. C., Adeli K. (2010). Fructose: a highly lipogenic nutrient implicated in insulin resistance, hepatic steatosis, and the metabolic syndrome. *American Journal of Physiology—Endocrinology and Metabolism*.

[20] Shi H., Cave B., Inouye K., Bjørbæk C., Flier J. S. (2006). Overexpression of suppressor of cytokine signaling 3 in adipose tissue causes local but not systemic insulin resistance. *Diabetes*.

[21] Gong F.-Y., Zhang S.-J., Deng J.-Y. (2009). Zinc-*α*2-glycoprotein is involved in regulation of body weight through inhibition of lipogenic enzymes in adipose tissue. *International Journal of Obesity*.

[22] Khitan Z., Harsh M., Sodhi K., Shapiro J. I., Abraham N. G. (2014). HO-1 Upregulation attenuates adipocyte dysfunction, obesity, and isoprostane levels in mice fed high fructose diets. *Journal of Nutrition and Metabolism*.

[23] Nadler S. T., Stoehr J. P., Schueler K. L., Tanimoto G., Yandell B. S., Attie A. D. (2000). The expression of adipogenic genes is decreased in obesity and diabetes mellitus. *Proceedings of the National Academy of Sciences of the United States of America*.

[24] Lan H., Rabaglia M. E., Stoehr J. P. (2003). Gene expression profiles of nondiabetic and diabetic obese mice suggest a role of hepatic lipogenic capacity in diabetes susceptibility. *Diabetes*.

[25] Previs S. F., Withers D. J., Ren J.-M., White M. F., Shulman G. I. (2000). Contrasting effects of IRS-1 versus IRS-2 gene disruption on carbohydrate and lipid metabolism in vivo. *The Journal of Biological Chemistry*.

[26] Zhao C. X., Xu X., Cui Y. (2009). Increased endothelial nitric-oxide synthase expression reduces hypertension and hyperinsulinemia in fructose-treated rats. *Journal of Pharmacology and Experimental Therapeutics*.

[27] Haas J. T., Miao J., Chanda D. (2012). Hepatic insulin signaling is required for obesity-dependent expression of SREBP-1c mRNA but not for feeding-dependent expression. *Cell Metabolism*.

[28] Alzamendi A., Giovambattista A., Raschia A. (2009). Fructose-rich diet-induced abdominal adipose tissue endocrine dysfunction in normal male rats. *Endocrine*.

[29] Alzamendi A., Giovambattista A., García M. E., Rebolledo O. R., Gagliardino J. J., Spinedi E. (2012). Effect of pioglitazone on the fructose-induced abdominal adipose tissue dysfunction. *PPAR Research*.

[30] Ma X., Lin L., Yue J. (2013). Ghrelin receptor regulates HFCS-induced adipose inflammation and insulin resistance. *Nutrition & Diabetes*.

[31] Rotter V., Nagaev I., Smith U. (2003). Interleukin-6 (IL-6) induces insulin resistance in 3T3-L1 adipocytes and is, like IL-8 and tumor necrosis factor-*α*, overexpressed in human fat cells from insulin-resistant subjects. *The Journal of Biological Chemistry*.

[32] Jager J., Grémeaux T., Cormont M., Le Marchand-Brustel Y., Tanti J.-F. (2007). Interleukin-1*β*-induced insulin resistance in adipocytes through down-regulation of insulin receptor substrate-1 expression. *Endocrinology*.

[33] Schneider K. S., Chan J. Y. (2013). Emerging role of Nrf2 in adipocytes and adipose biology. *Advances in Nutrition*.

[34] Enos R. T., Velázquez K. T., McClellan J. L., Cranford T. L., Walla M. D., Murphy E. A. (2014). Reducing the dietary omega-6: omega-3 utilizing a-linolenic acid; Not a sufficient therapy for attenuating high-fat-diet-induced obesity development nor related detrimental metabolic and adipose tissue inflammatory outcomes. *PLoS ONE*.

[35] Serrano-Marco L., Chacón M. R., Maymó-Masip E. (2012). TNF-*α* inhibits PPAR*β*/*δ* activity and SIRT1 expression through NF-*κ*B in human adipocytes. *Biochimica et Biophysica Acta—Molecular and Cell Biology of Lipids*.

[36] Magliano D. C., Penna-de-Carvalho A., Vazquez-Carrera M., Mandarim-de-Lacerda C. A., Aguila M. B. (2015). Short-term administration of GW501516 improves inflammatory state in white adipose tissue and liver damage in high-fructose-fed mice through modulation of the renin-angiotensin system. *Endocrine*.

[37] Barroso E., Rodríguez-Rodríguez R., Chacón M. R. (2015). PPAR*β*/*δ* ameliorates fructose-induced insulin resistance in adipocytes by preventing Nrf2 activation. *Biochimica et Biophysica Acta (BBA)—Molecular Basis of Disease*.

[38] Vasudevan H., Xiang H., McNeill J. H. (2005). Differential regulation of insulin resistance and hypertension by sex hormones in fructose-fed male rats. *American Journal of Physiology—Heart and Circulatory Physiology*.

[39] Vasudevan H., Nagareddy P. R., McNeill J. H. (2006). Gonadectomy prevents endothelial dysfunction in fructose-fed male rats, a factor contributing to the development of hypertension. *The American Journal of Physiology—Heart and Circulatory Physiology*.

[40] Kang W., Hong H. J., Guan J. (2012). Resveratrol improves insulin signaling in a tissue-specific manner under insulin-resistant conditions only: in vitro and in vivo experiments in rodents. *Metabolism*.

[41] Jimenez-Gomez Y., Mattison J. A., Pearson K. J. (2013). Resveratrol improves adipose insulin signaling and reduces the inflammatory response in adipose tissue of rhesus monkeys on high-fat, high-sugar diet. *Cell Metabolism*.

[42] Rivera L., Morón R., Zarzuelo A., Galisteo M. (2009). Long-term resveratrol administration reduces metabolic disturbances and lowers blood pressure in obese Zucker rats. *Biochemical Pharmacology*.

